# Long-term effect of full-body pulsed electromagnetic field and exercise protocol in the treatment of men with osteopenia or osteoporosis: A randomized placebo-controlled trial

**DOI:** 10.12688/f1000research.54519.2

**Published:** 2021-09-14

**Authors:** Anwar Ebid, Mohamed El-boshy, Shamekh El-Shamy, Ali Thabet, Mohamed Abedalla, Tariq Ali

**Affiliations:** 1 Physical Therapy, Umm Al-Qura University, Makkah, Saudi Arabia; 2 Laboratory Medicine, Umm Al-Qura University, Makkah, Saudi Arabia; 3 Internal Medicine, Ministry of Health KSA, Makkah, Saudi Arabia; 4 Umm Al-Qura University Medical Center, Umm Al-Qura University, Makkah, Saudi Arabia

**Keywords:** Pulsed electromagnetic field, Exercise protocol, bone mineral density, osteopenia, osteoporosis

## Abstract

**Background:** Osteoporosis is the most prevalent metabolic disease affecting bones. **Objective:** To investigate the long-term effect of pulsed electromagnetic field (PEMF) combined with exercise protocol on bone mineral density (BMD) and bone markers in men with osteopenia or osteoporosis. **Methods: **Ninety-five males with osteopenia or osteoporosis (mean age, 51.26 ± 2.41 years; mean height, 176 ± 2.02 cm; mean weight, 83.08 ± 2.60 kg; mean body–mass index (BMI), 26.08 ± 1.09 kg/m ^2^) participated in the study, and they were randomly assigned to one of three groups: Group 1 received a full-body PEMF and exercise protocol (PEMF +EX), Group 2 received a placebo full-body PEMF and exercise protocol (PPEMF +EX), and Group 3 received a full-body PEMF alone (PEMF). PEMF was applied for the whole body using a full-body mat three times per week for 12 weeks, with an exercise protocol that includes flexibility, aerobic exercise, strengthening, weight-bearing, and balance exercises followed by whole-body vibration (WBV) training. Outcome measures include BMD of total hip and lumbar spine and bone markers [serum osteocalcin (s-OC), Serum amino-terminal cross-linking telopeptide of type I collagen (s-NTX), Serum carboxy-terminal cross-linking telopeptide of type I collagen (s-CTX), Parathyroid hormones (PTH), Bone-specific Alkaline Phosphatase (BSAP), and 25-hydroxy vitamin D (Vit D)]. **Results: **The BMD of total hip and lumbar spine was significantly increased post-treatment in all groups, and more so in Group 1 and Group 2 than Group 3. There was a significant difference in bone markers in all groups, more so in Group 1 and Group 2 than in Group 3. **Conclusion:** PEMF combined with exercise protocol exerts a potent role for treating OP, is more effective than exercise and PEMF alone for increasing BMD and enhancing bone formation, and suppresses bone-resorption markers after 12-weeks of treatment with the impact lasting up to 6 months.

## Introduction

Osteoporosis (OP) is a systemic skeletal disease characterized by decreased bone mineral density, decreased bone quality, microarchitectural deterioration of bone tissue, and low bone mass, which causes discomfort, poor quality of life, increased bone fragility, and increased fracture risk, resulting in socio-economic burden, high morbidity and mortality, decreased functional mobility and increased attendant care and healthcare costs ( WHO Report, 2003; Christenson *et al*., 2012).

Pharmacological therapies, such as bisphosphonates, hormone replacement, raloxifene, calcium, parathyroid hormone (PTH), vitamin D, calcitonin, testosterone, and anabolic steroids, have all been used to treat OP in recent years, but long-term use of antiosteoporosis drugs can cause gastrointestinal problems, infections, jaw osteonecrosis, hypocalcemia, atypical subtrochanteric femoral fractures, increased risk of certain cancers, and atrial fibrillation ( Canalis *et al*., 2007; Body *et al*., 2012).

Biophysical stimulus employing physical therapy modalities, such as pulsed electromagnetic fields (PEMFs), light amplification by stimulated emission of radiation (LASER), and physical exercise, has been offered as alternative treatments that are less expensive, non-invasive, effective, safe, and causes fewer side effects, and is highly recommended for clinical use ( Wang *et al*., 2019). PEMFs are electromagnetic fields capable of producing biological currents in tissue and have unique biological effects. PEMFs also help patients with osteoporosis feel better by reducing pain, improving functional results and improving quality of life (QoL) ( Liu *et al*., 2013; Wang *et al*., 2019).

PEMFs may be one such effective therapy, and there is evidence that it has a positive impact on the treatment of various bone disorders, such as decreased bone mass, fresh fractures, non and delayed union, diabetic osteopenia, and osteonecrosis when compared with drug therapy ( Liu *et al*., 2013), through a variety of mechanisms including mechanical stimulation, regulating the proliferation, activity, and mineralization of bone marrow mesenchymal stem cells (BMSCs), as well as osteoblast proliferation, differentiation, and activity, as well as osteoclastogenesis and osteoclast differentiation. PEMF therapy has gained extensive use due to its quick effects, ease of use, and lack of side effects ( Liu *et al.*, 2013; Wang *et al*., 2019; Zhang *et al*., 2017; Li *et al*., 2014).

Exercise training, weight-bearing exercise, and strength training have been linked to the maintenance of bone mineral density (BMD) by enhancing and increasing the differentiation and activities of osteoblasts, which has a direct impact on the production of osteocalcin (OC), bone-specific alkaline phosphatase (BAP), 25-hydroxy vitamin D, and parathyroid hormones (PTH), which is more sensitive to exercise training and weight-bearing exercise ( Yuan *et al*., 2016; Mohammad *et al*., 2020).

Physical activity and mechanical effort increase mechanical signals, such as fluid flow, anabolic effect on osseous tissue dynamic tension, upregulate the expression of osteogenic markers compression, stimulate resident osteocytes through fluid shifts in their canalicular network, and stimulate biochemical markers of bone formation and hydrostatic pressure. These mechanical signals enhance osteogenic differentiation while inhibiting adipogenic differentiation, which could be one of the reasons why exercise prevents osteoporosis and improves bone health ( Maimoun and Sultan, 2009; Yuan *et al*., 2016).

Whole-body vibration (WBV) produces high-frequency mechanical stimulation that is distributed throughout the entire body. It has been proposed as a unique non-pharmacological method for the treatment of musculoskeletal problems ( Lau *et al*., 2011). WBV has been used as an alternate exercise strategy for bone and muscle stimulation. It has been demonstrated that increasing bone density *via* mechanical load and specific mechanical frequencies acting on the piezoelectric properties of bones can enhance osteogenesis, improve mechanical properties, accelerate fracture healing through angiogenesis, improve muscle function, increase BMD, reduce the risk of muscle power loss, improve muscle power, and help balance the musculoskeletal system ( Lau *et al*., 2011; Wong *et al*., 2021; Wang *et al*., 2017; Fratini *et al*., 2016).

To our knowledge, no previous study has investigated the combined effect of PEMF and exercise protocol on BMD and bone markers in men. Therefore, this research is to investigate the long-term effects of PEMF alone or in combination with an exercise protocol on BMD and bone markers in males with osteopenia or osteoporosis.

## Methods

### Ethical statement and trial registration

This was a three-measurement interval randomized placebo-controlled study. The Biomedical Research Ethical Committee of Umm Al-Qura University approved and considered this study ethically feasible (Approval number. HAPO-02-K-012-2021-540). The trial was also registered with the Clinical Trial Registry (Clinical Trials.gov ID: NCT04608162).

### Power analysis

The G-power for Windows was used to calculate the appropriate sample size, with estimated power = 0.95 and α = 0.05. Using analysis of variance (ANOVA), with in-between interaction in three groups and three measurement intervals, the effect size was 0.30. In all therapy groups, a minimum of 92 patients was recommended as a sample size.

### Participants

**Figure 1.**
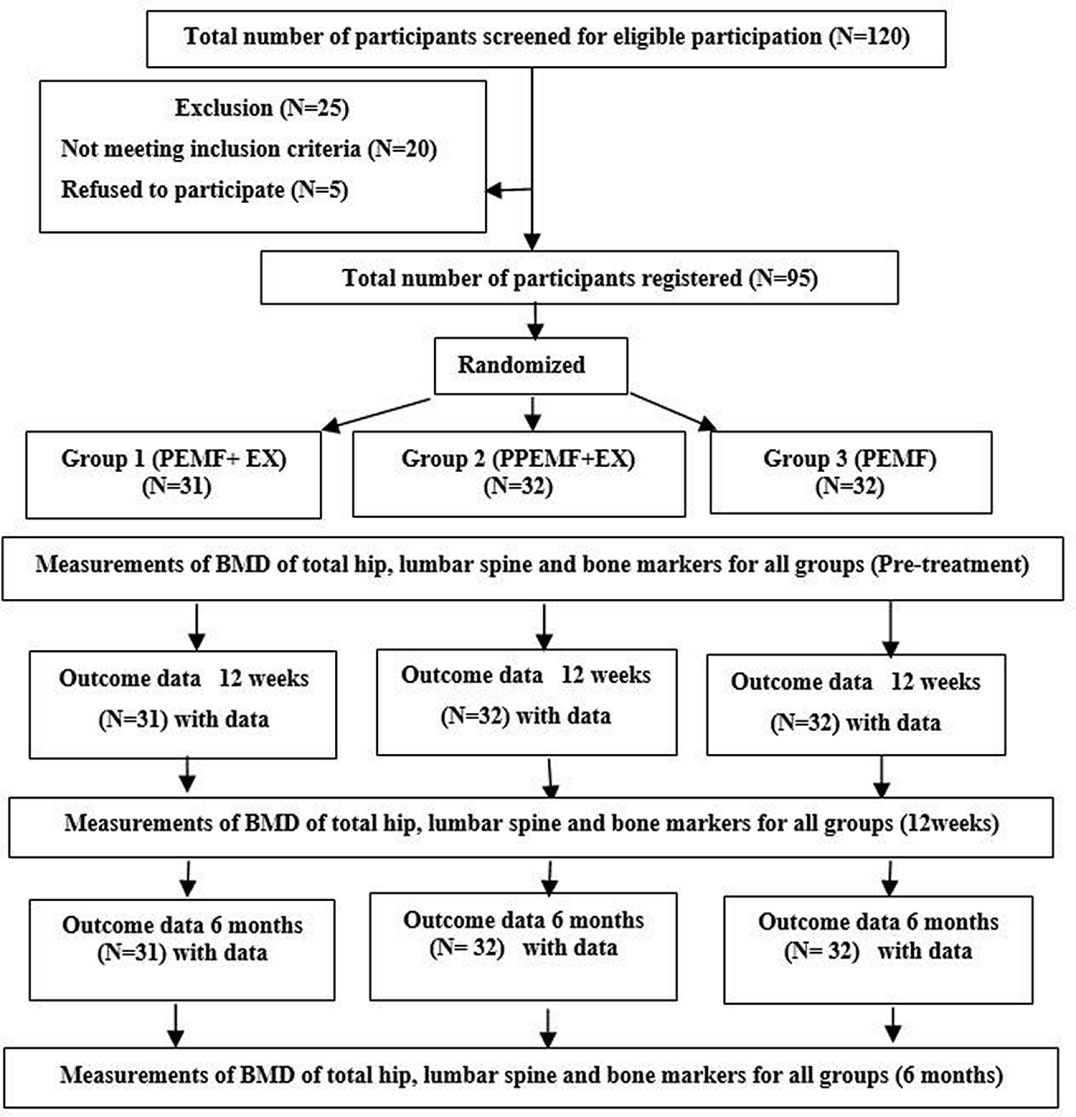
Flow chart of the study based on CONSORT criteria.

The study included 95 males over the age of 45 years (mean age, 51.26 ± 2.41 years; mean height, 176 ± 2.02 cm; mean weight, 83.08 ± 2.60 kg; mean body mass index (BMI), 26.08 ± 1.09 kg/m ^2^). Initially, all participants were assessed for the recruitment criteria. A medical and psychological history, including height, weight, and BMI, as well as physical and laboratory exams, were part of the screening process. Every participant was assessed physically to see how physically active they were. The presence of any gait issues, muscular soreness, or joint pain was considered during the exam. Any supplemental therapies, special diets, or aerobic exercise programs would not be allowed throughout the study if participants underwent physical therapy or a change in their pharmacological therapy during the previous 3 months. Throughout the trial, participants were asked to continue with their normal daily routines and to avoid any systematic exercise training regimens. By dual-energy X-ray absorptiometry (DEXA), all patients were diagnosed with osteopenia or osteoporosis (T-scores<−1.5). The same assessor, who was blind to treatment, assessed all participants at pre-treatment, at the end of 12 weeks of treatment, and at 6 months after the trial ended. To control for diurnal variability, follow-up was done at the same time of day as the baseline and after the 12-week evaluation, if practicable. The study goals were discussed after the pre-treatment evaluation, and all participants gave written informed consent for their participation and the publication of their study results. The flow diagram of the study depicts all of the study’s steps ( Figure 1).

Diabetes mellitus, intraocular lenses, thrombosis history, cardiopulmonary conditions, severe vascular and renal disease, uncontrolled thyroid disease, cardiac pacemaker, uncontrolled hypertension, progressive neurological disease, chronic disabling arthritis, use of any medications that affect bone metabolism, severe hepatic diseases, presence of osteoporotic fracture, significant anemia, neuropsychiatric disorders ( *e.g.*, dementia), alcohol abuse, severe depression, panic disorder, bipolar disorder, or psychosis and BMI <19 kg/m ^2^ or >31 kg/m ^2^ were all excluded from the study.

### Randomization

The randomization method was carried out with the use of an online GraphPad Prism (RRID:SCR_002798; R is an open access alternative **)** that divided the patients into three groups. Group 1 received full-body PEMF and an exercise protocol (PEMF+EX), while Group 2 received a placebo full-body PEMF and an exercise protocol (PPEMF+EX), and Group 3 received full-body PEMF alone (PEMF). After the initial evaluation of the participants, randomization was carried out, and they were blinded to the treatment group randomization. The external evaluator and therapists were blinded to the participant’s group allocation.

### DEXA evaluation

An OsteoSys PRIMUS densitometer (OsteoSys, Seoul, Korea) was used to assess BMD in all patients at the lumbar spine (L2–L4) and total hip pre-treatment, after 12 weeks of treatment and at 6-months as a follow-up. The Middle East (ethnicity) reference database, provided by the manufacturer, was used to calculate T-scores for detecting osteopenia and osteoporosis. Machine calibration was performed daily before the assessments using spine phantoms provided by the manufacturers. All measurements were made by the same operator for all patients throughout the study period.

### Bone markers

Serum osteocalcin (s-OC), serum amino-terminal cross-linking telopeptide of type I collagen (s-NTX), serum carboxy-terminal cross-linking telopeptide of type I collagen (s-CTX), bone-specific alkaline phosphatase (BSAP), parathyroid hormones (PTH), and 25-hydroxy vitamin D (Vit D) were collected by vein puncture into vacationer tubes with no additive and processed to serum, which was stored at −20°C until needed for analysis. All samples were analyzed in triplicate.

### Full-body pulsed electromagnetic field (FBPEMF) therapy

PEMF was applied to the whole body using a 68″×23″×1.5″ mat applicator and 8″×10″×2.5″ pillow applicator from Sedona Pro PEMF Systems, Portland Oregon USA Ltd). This mat generates a PEMF with a frequency range of 0.01–15,000 Hz, with sinusoidal, rectangular, multi-resonance, impulse, or sawtooth waveforms, the full-body maximum intensity is 30 gauss (3,000 microTesla) whereas the pillow is 101 gauss (10,000 microTesla). For a period of 12 weeks, each participant lay on the mat for 30 min/day, three times/week. The placebo intervention is identical to the actual intervention except that the PEMF device was not switched on. This method is particularly suitable for double-blind trials, as the application of PEMF therapy does not cause any feeling in the patient. The device used had a specially designed switch concealed at the back that enabled the independent researcher to interrupt the PEMF for the placebo group; the “on” sign and the parameters of PEMF therapy were displayed to all patients (placebo and PEMF groups) throughout the procedure.

### Exercise training protocol

The exercise protocol for each group consisted of three 60-min sessions per week for a total of 12 weeks, all of which were conducted in the exercise lab under the observation of the investigators. 40-min initial training exercises (flexibility, aerobic exercise, strengthening, weight-bearing, and balance exercises) were followed with WBV training exercise in each session. The initial training regimen was designed for beginning with a warm-up and gradually increased in intensity.

First, stretching exercises for the upper and lower limbs, as well as the back and abdominal muscles, were given to the participants. Treadmill walking exercises involve a 20-min warm-up at the lowest speed, followed by 10 min at an intensity of 40–60% of the predetermined personalized maximal heart rate and a 5-min cool-down at the lowest speed.

The treadmill walking was immediately halted if the subject displayed any signs of weariness, discomfort, balance disturbance, heavy perspiration, chest pain, dyspnea, or leg cramps. Strengthening exercises for the back extensor, hip abductor, flexor, and extensor, and knee extensor were performed after a 5-min break. Each exercise was done in sets of ten and repeated three times.

Back strengthening exercises were done on the bed against gravity, while hip strengthening activities were done in front of the wall bar in a weight-bearing position. Using a Total Gym gadget, a closed kinematic chain workout in the form of a leg press was conducted.

Three sets of 10 maximum repetitions each of isotonic resistance workouts of the hip muscles were then done using a sandbag with varied weights according to patient tolerance. Under the guidance of the therapist, jumping into position and stair climbing was done.

On a Power Plate pro 5 vibration platform, WBV training consisted of a high frequency (30–40 Hz) vibration stimulus at a modest setting (2–4 mm peak to peak) (Performance Health Systems, LLC, Northbrook, IL, USA). Each subject was exposed to vibrations while squatting in a static position.

For all participants, the foot positions were standardized. According to the theory of progressive overload, the training intensity was raised by decreasing the rest periods or increasing the amplitude and/or frequency of the vibration. The vibration exposure began with one set of 30 s at 2 mm amplitude and 30 Hz, progressively increasing to a final exposure at a high amplitude of 35 Hz with two sets of 5 min each. They took part in a cool-down phase at the end of the program, which included relaxation and stretching exercises.

Participants in all groups were encouraged to walk for 30 min each day. All participants were given detailed workout descriptions, and they kept track of their exercise compliance. If any person missed four consecutive exercise sessions, they were removed from the study.

### Outcome measures

All participants had their age (years), height (cm), weight (kg), and BMI (kg/m ^2^) measured, as well as BMD of the lumbar spine (L2–L4) and total hip. Bone markers such as s-OC, s-NTX, s-CTX, BSAP, PTH, and Vit D were also tested. Pre-treatment, 12-weeks post-treatment, and 6-month follow-up measurements were taken from all groups.

### Statistical analysis

G-Power 3.1 for Windows was used to calculate the estimated sample size based on the power analysis. One-way ANOVA was used to assess patient demographic data, such as age, weight, height, and BMI, using SPSS for Windows, version 16 ( **RRID:SCR_019096**); JASP ( **RRID:SCR_015823**) is an open-access alternative. ANOVA with a *post hoc* Bonferroni test was used to compare measurement intervals between treatment groups. Repeated measures ANOVA with *post hoc* Bonferroni test were used to measure baseline, after-treatment, and 6-month follow-up assessments in each group. For all tests, the level of significance was fixed at 0.05.

## Results

For this study, a total of 120 males were identified as potential participants ( Figure 1). Of these participants, 25 were excluded (20 not meeting inclusion criteria, and five refused to participate). A total of 95 males participated in this study and were randomized into three groups. Between the three groups, there were no significant variations in mean age, weight, height, or BMI ( Table 1). Following therapy, exercise compliance was 100% for all participants.

**Table 1.** Baseline values among treatment groups.

	Group 1 (PEMF+EX)	Group 2 (PPEMF+EX)	Group 3 (PEMF)	F value	P value
Age (years)	51.35 ± 2.56	51.87 ± 2.35	50.53 ± 2.15	2.790	0.0666 ^**^
Weight (kg)	82. 62 ± 2.95	82.96 ± 2.61	83.54 ± 2.11	1.031	0.3609 ^**^
Height (cm)	1.75 ± 2.18	1.76 ± 1.98	1.77 ± 1.26	0.0009	0.9991 ^**^
BMI (kg/cm ^2^)	27.00 ± 1.35	26.8 ± 1.32	26.7 ± 1.67	0.3454	0.7089 ^**^
Number of patients	31	32	32		
Number, osteopenic/osteoporotic hip (%) -1.1<T< -2.4	28 (90%)/3 (10%)	29 (91%)/3 (9%)	28 (88%)/4 (12%)		
Number, osteopenic/osteoporotic lumbar (%) -1.1<T< -2.4	30 (97%)/1 (3%)	31(97%)/1 (3%)	30 (94%)/2 (6%)		

** Non-significant differences in the same measurement interval among treatment groups (one-way ANOVA; p < 0.05).

BMI, body–mass index; EX, exercises; PEMF, pulsed electromagnetic field; PPEMF, placebo Pulsed electromagnetic field; p, probability value.

### BMD of the total hip

There were significant differences between the three groups at measurement intervals with a higher significance in the PEMF+Ex group than PPEMF+Ex and PEMF groups. In the PPEMF+EX group, there were significant differences between pre-treatment and 12-week values and between 12-week and 6-month values, but insignificant changes between pre-treatment and 6-month values ( Tables 2 and 3). Intergroup comparisons revealed significant differences between the 12-week and 6-month mean values ( Table 4).

**Table 2.** Changes in bone mineral density among treatment groups.

	Group 1 (PEMF+EX)			Group 2 (PPEMF+EX)			Group 3 (PEMF)			P value		
	Pre	12wk	6M	Pre	12wk	6M	Pre	12wk	6M	pre	12w	6M
Lumbar spine (BMD) Mean ± SD	0.99 ± 0.008	1.25 ± 0.20	1.32 ± 0.21	0.99 ± 0.006	1.09 ± 0.13	1.12 ± 0.14	0.99 ± 0.007	1.00 ± 0.03	0.98 ± 0.007	0.3718 ^**^	< 0.0001 ^*^	< 0.0001 ^*^
P value	< 0.0001 ^*^			< 0.0001 ^*^			0.0002 ^*^					
F value	33.43			11.80			9.53			1.000	36.87	63.47
Total hip (BMD) Mean ± SD	0.92 ± 0.008	0.94 ± 0.02	0.94 ± 0.007	0.92 ± 0.01	0.93 ± 0.014	0.93 ± 0.015	0.92 ± 0.013	0.93 ± 0.015	0.91 ± 0.012	0.3718 ^**^	0.0251 ^*^	< 0.0001 ^*^
P value	< 0.0001 ^*^			0.0063 ^*^			< 0.0001 ^*^					
F value	107.8			5.98			17.83			1.000	3.385	52.57

* Significant difference in the same measurement interval among treatment groups (one-way ANOVA; p < 0.05).

* Significant difference among the repeated measurement intervals in each treatment group (repeated measures ANOVA; p < 0.05).

** Non-significant differences.

12W, 12weeks; 6M, 6 Months; BMD, bone mineral density; SD, standard deviation.

**Table 3.** Comparison between measurements in each treatment group.

	Total hip (BMD)			Lumbar spine (BMD)		
	P value			P value		
	Pre vs 12w	Pre vs 6 M	12W vs 6 M	Pre vs 12w	Pre vs 6 M	12W vs 6 M
PEMF+EX	< 0.0001 *	< 0.0001 *	0.0156 *	< 0.0001 *	< 0.0001 *	0.5807 **
PPEMF+EX	0.0017 *	> 0.0999 **	0.0017 *	< 0.0001 *	< 0.0001 *	0.0017 *
PEMF	0.0059 *	0.0022 *	< 0.0001 *	0.0711 **	< 0.0001 *	< 0.0001 *

* Significant difference in the same measurement interval among treatment groups (post hoc Bonferroni test; p < 0.05).

** Non-significant differences.

**Table 4.** Comparison between each measurement interval among treatment groups.

	Total hip (BMD)			Lumbar spine (BMD)		
	P value			P value		
	Pre	12wk	6M	Pre	12wk	6M
PEMF+EX vs PPEMF+EX	> 0.0999 ^**^	0.0246 ^*^	0.0002 ^*^	> 0.0999 ^**^	0.0004 ^*^	0.0014 ^*^
PEMF+EX vs PEMF	> 0.0999 ^**^	< 0.0001 ^*^	< 0.0001 ^*^	> 0.0999 ^**^	< 0.0001 ^*^	< 0.0001 ^*^
PPEMF+EX vs PEMF	> 0.0999 ^**^	0.0018 ^*^	0.0007 ^*^	> 0.0999 ^**^	< 0.0001 ^*^	0.0002 ^*^

* Significant difference in the same measurement interval among treatment groups; p < 0.05).

** Nonsignificant differences.

### BMD of the lumbar spine

In all three groups, there were significant intragroup differences among the measurement intervals. The significance was higher in the PEMF+Ex group than PPEMF+Ex and PEMF groups. In the PEMF+EX group, there were significant differences between pre-treatment and 12-weeks or 6-months as follow-up values, but insignificant changes between 12-weeks and 6-months. In the PEMF group, there were significant differences between pre-treatment and 6- months and 12-weeks and 6-months values, but insignificant changes between pre-treatment and 12-weeks ( Tables 2 and 3). Intergroup comparisons revealed significant differences in 12-weeks and 6-months mean values ( Table 4).

### Bone markers

Serum s-OC revealed a significant decrease at 12-weeks in PEMF+EX and PPEMF+EX groups when compared with the pre-treatment values and PEMF group, but no significant difference in the PEMF group. At 12-weeks, there was no significant difference between the PEMF+EX and the PPEMF+EX groups. When comparing the PEMF+EX group with the PPEMF+Ex and PEMF groups at 6-months, there was a substantial decrease in the level of s-OC in the PEMF+EX group ( Table 5). When compared with pre-treatment values and the PEMF group, the s-NTX and s-CTX in PEMF+EX and PPEMF+EX exhibited a substantial decrease at 12-weeks, while the PEMF group showed no significant difference. When comparing PEMF+EX with PPEMF+EX and PEMF groups at 12 weeks, there was a significant difference; furthermore, when comparing PPEMF+EX to the PEMF group at 12-weeks, there was a significant difference. When comparing the PEMF+EX group to the PPEMF+Ex and PEMF groups at 6-months, the level of s-NTX and s-CTX was significantly lower in the PEMF+EX group ( Table 5).

**Table 5.** Changes in bone markers among treatment groups.

Parameters	PEMF+EX			PPEMF+EX			PEMF		
	Pre	12wk	6M	Pre	12wk	6M	Pre	12wk	6M
s-OC (ng/ml)	24.05 ± 6.12	18.42 ± 3.61 * ^b^	20.35 ± 4.21 ^b^	24.67 ± 6.32	19.67 ± 3.61 ^b^	22.50 ± 6.46 ^ab^	22.82 ± 6.31	22.01 ± 6.17 ^a^	25.05 ± 7.32 ^a^
P value (F value)	< 0.0001 ^*^ (11.16)			0.0025 ^*^ (6.37)			0.08590 ^**^ (0.1522)		
s-NTX (nmol/L)	25.75 ± 7.01	14.15 ± 5.02 * ^c^	16.77 ± 4.16 ^c^	25.82 ± 7.30	21.20 ± 5.96 * ^b^	23.05 ± 5.92 ^b^	26.80 ± 6.87	25.25 ± 7.09 ^a^	26.55 ± 7.33 ^a^
P value (F value)	< 0.0001 ^*^ (37.56)			0.0181 * (4.191)			0.6456 * * (0.4397)		
s-CTX (Pg/ml)	639 ± 57.62	455 ± 44.31 * ^c^	544 ± 94.31 ^b^	629 ± 50.88	496 ± 66.54 * ^b^	627 ± 48.41 ^a^	631 ± 48.43	615 ± 72.21 ^a^	633 ± 46.48 ^a^
P value (F value)	< 0.0001 ^*^ (55.54)			< 0.0001 ^*^ (59.58)			0.3862 ^**^(0.9613)		
BSAP (U/L)	45.65 ± 11.42	24.05 ± 6.75 * ^c^	28.55 ± 6.53 ^c^	47.01 ± 10.47	30.16 ± 10.14* ^b^	33.43 ± 9.71 ^b^	47.98 ± 12.04	41.18 ± 9.98* ^a^	48.07 ± 9.12 ^a^
P value (F value)	< 0.0001 ^*^ (55.25)			< 0.0001 ^*^ (24.99)			0.0127 ^*^ (4.575)		
PTH (pg/mL)	33.85 ± 5.10	32.27 ± 5.30* ^c^	34.35 ± 3.91 ^a^	34.87 ± 3.25	34.65 ± 2.97 ^a^	34.81 ± 4.08 ^a^	34.70 ± 3.55	34.57 ± 3.49 ^b^	34.52 ± 3.65 ^a^
P value (F value)	0.2116 ^**^(1.580)			0.9661 ^**^ (0.0344)			0.9785 ^**^(0.0217)		
25(OH) VD (ng/mL)	26.65 ± 6.37	26.52 ± 6.52 ^a^	27.12 ± 6.79 ^a^	27.91 ± 6.27	27.52 ± 6.16 ^a^	25.74 ± 6.97 ^a^	26.80 ± 6.06	27.07 ± 6.17 ^a^	27.01 ± 6.85 ^a^
P value (F value)	0.0620 ^**^ (0.9399)			0.3643 ^**^ (1.021)			0.0158 ^**^ (0.9843)		

* Significant difference in each treatment group (one-way ANOVA; p < 0.05).

** Non-significant differences.

The same letter is non-Significant difference between groups.

s-OC; Serum osteocalcin; s-NTX; Serum amino-terminal cross-linking telopeptide of type I collagen; s-CTX; Serum carboxy-terminal cross-linking telopeptide of type I collagen; BSAP, Bone Serum Alkaline Phosphatase; PTH, Parathyroid hormone; 25(OH) vit D; 25-hydroxy vitamin D.

The BSAP in all groups reduced significantly after 12-weeks as compared to pre-treatment levels and the PEMF group. When compared to PPEMF+EX and PEMF groups at 12 weeks, PEMF+EX showed a significant difference; additionally, PPEMF+EX showed a significant difference when compared to the PEMF group at 12 weeks. When comparing the PEMF+EX group to the PPEMF+Ex and PEMF groups at 6-months, the level of B-SAP was significantly lower in the PEMF+EX group. Within and between groups, there were no significant variations in serum PTH and 25(OH) VD levels ( Table 5).

## Discussion

### Effect of PEMF

The proposed mechanisms of PEMF’s impact on bone, particularly in OP, are unlikely to be detailed in the literature, and more research into the mechanism of action and effect on osteoporosis is needed. PEMF has recently gained popularity as a treatment option for musculoskeletal issues, such as pain, inflammation, tissue regeneration, osteopenia, osteoporosis, and bone healing. As a result, we investigated the impact of PEMF alone or in combination with exercise protocol on osteopenia or osteoporosis and bone markers. Our major findings demonstrate that PEMF, alone or in combination with an exercise protocol, has a significant influence on BMD of the hip and lumbar spine, bone-formation, and bone-resorption markers.

PEMF has been shown to enhance osteogenesis, prevent bone loss, increase BMD, improve fracture healing, and enhance osteoblast activity, resulting in increased cell differentiation in both experimental and therapeutic settings ( Sert *et al*., 2000; Shen and Zhao, 2010; Androjna *et al*., 2014; Liu *et al*., 2013). PEMF therapy also increased the levels of biomarkers of osteoblast-associated bone formation, such as serum bone-specific alkaline phosphatase (BSAP), serum osteocalcin (OC), and serum carboxy-terminal propeptide of type I collagen (PINP), while decreasing the levels of serum C-terminal telopeptide (CTX), which was independent of BMD change ( Jing *et al*., 2014; Giordano *et al*., 2001; Spadaro *et al*., 2011).

PEMFs’ biological activity could be linked to the amplification mechanisms that take place during transmembrane coupling ( Ciombor and Aaron, 2005). Amplification is most likely to occur at transmembrane receptors. Several membrane receptors and pathways, including the PTH pathway, insulin, insulin-like growth factor (IGF-2), and calcitonin ( Jiang *et al*., 2016), have been found to be affected by PEMFs in terms of ligand binding and distribution as well as activity. As a result, transmembrane signaling is modulated ( Hu *et al*., 2020). PEMFs have a considerable anti-inflammatory and analgesic effect on the joint environment ( Varani *et al*., 2017).

In multiple studies, PEMFs have been shown to considerably reduce pain and enhance the quality of life in individuals with primary OP ( Spadaro *et al*., 2011). PEMFs can also considerably downregulate biomarkers associated with bone resorption as well as upregulate biomarkers associated with bone growth, as well as have also been confirmed to improve BMD in the distal radius, spine, and knees of patients with OP ( Roozbeh *et al*., 2018).

In a recent analysis, various underlying molecular signaling pathways of PEMFs mechanism of action were summarized on bone repair, including Ca2+, Wnt/β-catenin, mitogen-activated protein kinase (MAPK), fibroblast growth factor (FGF), vascular endothelial growth factor (VEGF), transforming growth factor (TGF)-β ( Lohmann *et al*., 2003), bone morphogenetic proteins (BMP) ( Aron *et al*., 2002), IGF, Notch, Prostaglandin E2 (PGE2) ( Holmes, 2017), and cAMP/protein kinase A (PKA) ( Miyamoto *et al*., 2019). Furthermore, it has been shown that the mammalian target of the rapamycin (mTOR) pathway is the underlying signaling mechanism of PEMFs implicated in bone formation ( Fitzsimmons *et al*., 1992) and boosting the production of extracellular matrix (ECM) proteins and enabling tissue healing ( Ciombor and Aaron, 2005).

Despite the favorable effects of PEMFs on bone mineral density (BMD) in patients with OP, the results of some studies remain controversial, as a single-blind, randomized pilot trial found no significant improvement in BMD ( Giordano *et al.,* 2001). Furthermore, a randomized, sham-controlled trial found no long-term substantial favorable effects of PEMFs on BMD in patients with forearm disuse osteopenia ( Spadaro *et al*., 2011). Similarly, following an 8-year follow-up ( Tabrah *et al*., 1998), there were no further favorable effects of PEMFs (72 Hz, 10 h each day of 12 weeks) on BMD. The contradictory results could be attributable to the limited sample size of this research, as well as the fact that different groups used various clinical characteristics and strategies ( Wang *et al*., 2019).

### Effect of exercise and WBV

In this study, after 12-weeks of treatment and 6-months of follow-up, an exercise protocol significantly increased lumbar and total hip BMD, enhanced bone formation, and suppressed bone resorption markers in males with osteopenia or osteoporosis. The current study’s findings were in line with those of earlier clinical trials ( Maddalozzo and Snow, 2000; Kukuljan *et al*., 2009; Almstedt *et al*., 2011; Bemben and Bemben, 2011; Kukuljan *et al*., 2011; Alayat *et al*., 2018) but not with those of other investigations ( Huuskonen *et al*., 2001; Woo *et al*., 2007; Whiteford *et al*., 2010). The contrast between the current study findings and earlier studies could be explained by differences in study design, duration, age ranges, as well as exercise type and intensity ( Huuskonen *et al*., 2001; Woo *et al*., 2007; Whiteford *et al*., 2010).

Exercise and strength training have been linked to bone mineral density (BMD) maintenance by increasing osteoblastic activity, which has a direct effect on OC production (bone remolding marker and osteoblast-specific protein) but also acts as an active hormone which responsible for the manner in which bone, adipose tissue, and muscle cross-communicate and how they impact glucose homeostasis in humans and has an important role in metabolic signaling in skeletal muscle and bone, and improves insulin sensitivity ( Mohammad *et al*., 2020; Villareal *et al*., 2006).

Exercise has been found to alter calciotropic hormones, vitamin D, and parathormone (PTH), all of which are significant regulators of bone metabolism. In recent studies, a brief bout of exercise temporarily enhanced PTH secretion, which plays a variety of roles in bone turnover ( Maimoun and Sultan, 2009), treatment of osteoporosis with an occasionally delivered PTH analog, on the other hand, has been demonstrated to boost bone production indicators and BMD ( Neer *et al*., 2001).

WBV exercise has been widely recommended for prevention and treatment of osteoporosis and increasing BMD, thereby improving bone mass, strength, and reducing bone destruction ( Totosy de Zepetnek *et al*., 2009; Lau *et al*., 2011), and it appears to be a safe, non-invasive, non-pharmacological intervention and effective training method for maintaining or improving bone metabolism in a variety of group populations. Thus, the WBV modality may be an ideal approach to osteoporosis treatment for some specific populations ( Bemben *et al*., 2010; Wong *et al*., 2021).

Mechanical signals, a major component of exercise, increase mesenchymal stem cells (MSC), activate mechanotransduction in bone, osteogenesis, proliferation, and reduce inflammatory marker levels when given in the form of low-intensity vibration ( Bas *et al*., 2020). The direct transmissibility of vibratory signals to bone cells, resulting in osteogenic responses, is hypothesized by ( Judex and Rubin, 2010) as a viable mechanism by which WBV training can generate anabolic or anti-catabolic responses in bone tissue. Rubin *et al*. (2004) found a significant difference in BMD change between the placebo and experimental groups after WBV training and conclude that WBV training can help to maintain and improve BMD ( Rubin *et al*., 2004).

Recently, there is growing evidence that exercise training leads to maintain and raised the level of undercarboxylated osteocalcin (uc-OCN), leptin (which plays an essential role in bone formation), and glucose homeostasis and adiponectin (which contribute to bone metabolism by increasing glucose utilization and fatty acid oxidation) ( Mohammad *et al*., 2020; Levinger *et al*., 2017; Hiam *et al*., 2020).

Whole-body vibration (WBV) alone or in combination with multi-component exercise programs of strength, aerobic, high impact, and/or weight-bearing training, as well as whole-body vibration (WBV) alone or in combination with exercise protocol, may help to improve functional mobility, QoL, and depressive symptoms in post-menopausal women, adults, and older populations. ( Gomez-Cabello *et al*., 2012; Bolam *et al*., 2013; Sen *et al*., 2020). According to ( Jepsen *et al*., 2019), adding WBV to teriparatide resulted in a significant increase in BMD in the lumbar spine at 12 months, as well as indices of bone turnover after 3 and 6 months.

## Conclusions

PEMF combined with exercise protocol was more effective than exercise and PEMF alone at increasing hip and lumbar BMD and have a beneficial effect on bone markers after 12-weeks of treatment, with effects lasting up to 6 months.

## Recommendation

The effects of such combinations should be investigated in other areas, such as the cervical or forearm, and for longer periods of time, as well as in osteoporosis-affected women.

## Study strength and limitations

Although there have been numerous studies evaluating the efficacy of PEMF either alone or with a combination of different exercise types in different groups of populations, there have been few studies evaluating the long-term efficacy of PEMF alone or combined with exercise protocol on male osteoporosis. The use of an exercise protocol in our prospective, randomized, controlled study allowed us to assess the long-term efficacy of the PEMF and exercise protocol compared with placebo PEMF and to compare the effects of the PEMF and exercise protocol to each other. In the literature review, there were no similar studies comparing PEMF and exercise protocol on BMD and bone markers in males. Another strength of this study was that the interventions were implemented as supervised group exercises. In the follow-up evaluations, in addition to measuring the BMD values, the use of various evaluation parameters, such as the determination of the bone turnover markers levels, can be listed as additional factors that strengthened our study. There were a few limitations to our study that should be noted when interpreting the data. For starters, our study’s follow-up time was just 6 months, which was insufficient to establish the efficacy of PEMF and existing exercise protocol on BMD or bone turnover markers. Previous research has revealed that it takes a long time for bone metabolism to produce an exercise response. Second, all study participants were instructed to maintain a well-balanced diet and engage in a regular exercise plan at home, which comprised 30 min of daily walking and tracking their exercise compliance. Because none of the participants reported any drawbacks, serious adverse events, or new-onset local pain, we regarded the home-prescribed exercise program to be a limiting factor in this study.

## Data Availability

Figshare: Underlying data for ‘Long-term effect of full-body pulsed electromagnetic field and exercise protocol in the treatment of men with osteopenia or osteoporosis: A randomized placebo-controlled trial’. The project contains the following underlying data:
• Age, height and weight raw data: https://doi.org/10.6084/m9.figshare.14915805
• BMD total hip and lumbar spine raw data: https://doi.org/10.6084/m9.figshare.14916057
• Bone markers raw data: https://doi.org/10.6084/m9.figshare.14916078 Figshare: Extended data for ‘Long-term effect of full-body pulsed electromagnetic field and exercise protocol in the treatment of men with osteopenia or osteoporosis: A randomized placebo-controlled trial’. The project contains the following underlying data:
• Flow chart of the study: https://doi.org/10.6084/m9.figshare.14915781
• Tables: https://doi.org/10.6084/m9.figshare.14915790
• CONSORT Checklist: https://doi.org/10.6084/m9.figshare.14916261 Data are available under the terms of the Creative Commons Zero “No rights reserved” data waiver (CC0 1.0 Public domain dedication).
