# Supplementary material for: Long-term effect of full-body pulsed electromagnetic field and exercise protocol in the treatment of men with osteopenia or osteoporosis: A randomized placebo-controlled trial
Source: F1000Res. 2021 Nov 29;10:649. Originally published 2021 Jul 26. [Version 3] doi: 10.12688/f1000research.54519.3 (PMC8637238; doi:10.12688/f1000research.54519.3)
Supplement: Peer review history

# Peer review: Reviewer response for version 3

**DOI:** 10.5256/f1000research.79642.r101430

**Published:** 2021-12-01

**Authors:** Mohamed Taher Ahmed Omar

**Affiliations:** 1 Rehabilitation Health Sciences, College of Applied Medical Sciences, King Saud University, Riyadh, Saudi Arabia

Thank you to the authors for their hard work especially in making the requested adjustments.

Is the work clearly and accurately presented and does it cite the current literature?

Yes

If applicable, is the statistical analysis and its interpretation appropriate?

Yes

Are all the source data underlying the results available to ensure full reproducibility?

Yes

Is the study design appropriate and is the work technically sound?

Yes

Are the conclusions drawn adequately supported by the results?

Yes

Are sufficient details of methods and analysis provided to allow replication by others?

Yes

Reviewer Expertise:

physical modalities in rehabiltation, outcome measures , onclogy rehab

I confirm that I have read this submission and believe that I have an appropriate level of expertise to confirm that it is of an acceptable scientific standard.

# Peer review: Reviewer response for version 2

**DOI:** 10.5256/f1000research.76679.r98634

**Published:** 2021-11-15

**Authors:** Mohamed Taher Ahmed Omar

**Affiliations:** 1 Rehabilitation Health Sciences, College of Applied Medical Sciences, King Saud University, Riyadh, Saudi Arabia

The authors have collected a unique dataset using sound methodology. The paper is generally well written and structured.  The methodology was clearly described and well defined and responded to the set objectives, with using bone mineral density as the gold standard for diagnosing osteoporosis and the best quantitative indicator for forecasting the risk of osteoporotic fracture, monitoring

**Abstract:**

- Generally, I suggest deleting the numerical values related to weight, height, and BMI and adding data related to PEMF setting parameters such as treatment duration, frequency, intensity.
- No information was provided about balance exercises in the text, so I suggest deleting it from the abstract.
- I would prefer to add some numerical values / as well as p-values of reported outcome measures in the result section.
- Add more information regarding significance across duration (pre, 12 weeks, and 6 months)
- In conclusion; OP stands for what e.g. osteoporosis/ osteopenia, PEMF changed to PPEMF

**Introduction: **The overall structure of the introduction is well-organized and well-balanced. However, some grammatical and more editing is required.

- **Paragraph 1, line 1; **I suggest changing “Osteoporosis (OP) is a systemic skeletal disease” to Osteoporosis (OP) is a systemic metabolic bone disease. In general; I suggest rewriting this paragraph, as poor quality of life (QoL) is the end result of the disease-related effect

**Methods:**

- Power analysis should be written under the statistical analysis section
- numerical values related to age, weight, height, and BMI and add data should be reported under the results section.

**Participants:**

- Data such as “height, weight, and BMI, as well as physical and laboratory exams not considered as medical and psychological as author reported, but as sociodemographic and medical; please check and correct.
- How author measures and determine the level of physical activity, for example, using self-reported questionnaire, or performed based measures, and how the author classifies patients according to this finding e.g. sedentary life, physically active, and physically inactive; please explain.
- Please, rewrite this statement to be matched with methodology “Any supplemental therapies, special diets, or aerobic exercise programs would not be allowed throughout the study" (how the patients would not be allowed to aerobic exercises throughout the study), according to the authors the aerobic exercises are part of exercises protocol implemented in the current study.
- Be consistent using either term patients/ participants/ subjects
- Delete the word “if practicable”
- Please check exclusion criteria and remove duplicated and redundancy such as thrombosis history/ severe vascular, cardiopulmonary conditions/cardiac pacemaker how therapist excluded all these disorders, e.g. face to face interview and physical examination, if yes, how this examination was done, through direct contact with a physician, and/or check-in medical records, please explain.
- Please provide more data about PEMF parameters setting such as duration of treatment, frequency, flux intensity.
- Please provide more information about randomization methods for example block randomization, stratified randomization.

**Exercises training protocol:**

- Treadmill walking exercises involve a 20-min warm-up at the lowest speed is this statement correct or is the reported duration for aerobic exercises as a whole.
- Please explain, who determines the intensity of 40–60% of the predetermined personalized maximal.
- Please explain the way of exercises protocol progress during 12 weeks interval 

**Outcome measures: **

- Who this variable considers as outcome measures “All participants had their age (years), height (cm), weight (kg), and BMI (kg/m2) measured, “

**Statistical analysis:**

- Delete duplicated statements about power analysis.
- Did the author evaluate the normality of the data? the author described the demographic data based on the results of normal analysis.

**Results:**

- I would like if the authors can change the title of table 1 to *Table 1: Participant characteristics (n=95)*
- No statistical test/values are reported in table 1 for Number, osteogenic/ osteoporotic hip, and lumber; please explain.
- Please check the significance of p values in the footnote under table 1.
- Please add numerical values for each group as Group 1 (PEMF+EX) (n=31) and delete this row from table 2.
- I suggest merging data reported in table 3, and 4  to be included in table 2. Table 2 p-values reported before f-value, please rearrange.
- it is suggested to measure the effect size of different interventions as significant differences did not reflect clinically significant, as the effect size is one of the most important indicators of clinical significance.

**Discussion:**

- **Paragraph 4;** I suggest deleting this statement **“** **In multiple studies, PEMFs have been shown to considerably reduce pain and enhance the quality of life in individuals with primary OP (Spadaro** **et al** **., 2011).** ****As the author did not evaluate or measures pain and quality of life

Is the work clearly and accurately presented and does it cite the current literature?

Yes

If applicable, is the statistical analysis and its interpretation appropriate?

Yes

Are all the source data underlying the results available to ensure full reproducibility?

Yes

Is the study design appropriate and is the work technically sound?

Yes

Are the conclusions drawn adequately supported by the results?

Yes

Are sufficient details of methods and analysis provided to allow replication by others?

Yes

Reviewer Expertise:

physical modalities in rehabiltation, outcome measures , onclogy rehab

I confirm that I have read this submission and believe that I have an appropriate level of expertise to confirm that it is of an acceptable scientific standard, however I have significant reservations, as outlined above.

# Response

**Published:** 2021-11-19

**Authors:** Anwar Ebid

**Affiliations:** Umm-Al-Qura University, Saudi Arabia

Dear Prof. Mohamed Taher,

 Thank you very much for taking the time to write a review.

 The authors are grateful for the reviewers' volunteer contributions in the form of helpful comments that have helped us strengthen our article. The authors have made a concerted effort to update the paper in light of your suggestions.

 We've updated the article in response to your feedback.

 Kindly check these updated versions as the article's revised version.

 Thank you.

 Regards,

 Authors

*Approved With Reservations*

*The authors have collected a unique dataset using sound methodology. The paper is generally well written and structured.  The methodology was clearly described and well defined and responded to the set objectives, with using bone mineral density as the gold standard for diagnosing osteoporosis and the best quantitative indicator for forecasting the risk of osteoporotic fracture, monitoring*

*Abstract:*

*Generally, I suggest deleting the numerical values related to weight, height, and BMI and adding data related to PEMF setting parameters such as treatment duration, frequency, intensity.*

**Reply: Numerical values for weight, height and BMI were deleted, and parameters for PEMF were added.**

*No information was provided about balance exercises in the text, so I suggest deleting it from the abstract.*

**Reply: Balance exercises were deleted from the abstract and methodology sections.**

*I would prefer to add some numerical values / as well as p-values of reported outcome measures in the result section.*

**Reply: The maximum number of words in the abstract, according to the journal guidelines for writing a research article, is 300 words, so adding the numerical values and p values of reported outcome measures will exceed the maximum number of words in the abstract, and all data of outcome measures represented and tabulated in the text under the result section.**

     

*Add more information regarding significance across duration (pre, 12 weeks, and 6 months)*

**Reply: The maximum number of words in the abstract, according to the journal guidelines for writing a research article, is 300 words, so adding the numerical values and p values of reported outcome measures will exceed the maximum number of words in the abstract, and all data of outcome measures represented and tabulated in the text under the result section.**

     

*In conclusion; OP stands for what e.g. osteoporosis/ osteopenia, PEMF changed to PPEMF*

**Reply: OP related to osteoporosis.**

*Introduction: The overall structure of the introduction is well-organized and well-balanced. However, some grammatical and more editing is required.*

*Paragraph 1, line 1; I suggest changing “Osteoporosis (OP) is a systemic skeletal disease” to Osteoporosis (OP) is a systemic metabolic bone disease. In general; I suggest rewriting this paragraph, as poor quality of life (QoL) is the end result of the disease-related effect.*

**Reply: The paragraph was re-written.**

*Methods:*

*Power analysis should be written under the statistical analysis section*

**Reply: Power analysis was transferred to the statistical analysis section.**

     

*numerical values related to age, weight, height, and BMI and add data should be reported under the results section.*

**Reply: Numerical values related to age, weight, height, and BMI were reported under the results section.**

*Participants:*

*Data such as “height, weight, and BMI, as well as physical and laboratory exams not considered as medical and psychological as author reported, but as sociodemographic and medical; please check and correct.*

**Reply: The paragraph was corrected in the text.**

     

*How author measures and determines the level of physical activity, for example, using a self-reported questionnaire, or performed based measures, and how the author classifies patients according to this finding e.g., sedentary life, physically active, and physically inactive; please explain.*

**Reply: The participants in the study were selected via a self-reported questionnaire that included sociodemographic data, physical characteristics, and medical information, and the participants were included in the study based on the analysis of these data.**

    

*Please, rewrite this statement to be matched with methodology “Any supplemental therapies, special diets, or aerobic exercise programs would not be allowed throughout the study" (how the patients would not be allowed to aerobic exercises throughout the study), according to the authors the aerobic exercises are part of exercises protocol implemented in the current study*.

**Reply: The statement was corrected in the text.**

*Be consistent using either term patients/ participants/ subjects*

**Reply: The term participants throughout the manuscript.**

*Delete the word “if practicable”*

**Reply: This word has been deleted from the text.**

 Plea *se check exclusion criteria and remove duplicated and redundancy such as thrombosis history/ severe vascular, cardiopulmonary conditions/cardiac pacemaker how therapist excluded all these disorders, e.g. face to face interview and physical examination, if yes, how this examination was done, through direct contact with a physician, and/or check-in medical records, please explain.*

**Reply: The content has been edited to remove the duplicated words. Because one of the co-authors is a physician, the exclusion was done through medical records check-in and examination.**

     

*Please provide more data about PEMF parameters setting such as duration of treatment, frequency, flux intensity.*

**Reply: The data about PEMF parameters setting was added to the text in the methodology section.**

*Please provide more information about randomization methods for example block randomization, stratified randomization.*

**Reply: The study's randomization approach was stratified randomization.**

*Exercises training protocol: Treadmill walking exercises involve a 20-min warm-up at the lowest speed is this statement correct or is the reported duration for aerobic exercises as a whole.*

**Reply: The reported duration for aerobic exercises as a whole**

     

*Please explain, who determines the intensity of 40–60% of the predetermined personalized maximal.*

**Reply: The individuals' age-related personalized maximum heart rate was calculated by the study's teamwork using the simple formula (220- Age).**

     

*Please explain the way of exercises protocol progress during 12 weeks interval*

**Reply: The exercise protocol in our study stayed constant throughout the duration of the study.**

*Outcome measures: Who this variable considers as outcome measures “All participants had their age (years), height (cm), weight (kg), and BMI (kg/m2) measured “*

**Reply: The outcome measures are edited in the text, and these variables are considered demographic data.**

*Statistical analysis:  Delete duplicated statements about power analysis.*

**Reply: In the text, the statement has been edited.**

    

*Did the author evaluate the normality of the data? the author described the demographic data based on the results of normal analysis.*

**Reply: Yes, data normalization was performed.**

*Results: I would like if the authors can change the title of table 1 to Table 1: Participant characteristics (n=95)*

**Reply: In the text, the title of table 1 has been changed.**

     

*No statistical test/values are reported in table 1 for Number, osteogenic/ osteoporotic hip, and lumber; please explain.*

**Reply: By dividing the number by the total and multiplying by 100, the percentage of osteogenic/osteoporotic in the hip and lumbar was calculated.**

   

*Please check the significance of p values in the footnote under table 1*.

**Reply: The footnote under table 1 was checked**

     

*Please add numerical values for each group as Group 1 (PEMF+EX) (n=31) and delete this row from table 2.*

**Reply: Table 2 was modified.**

     

*I suggest merging data reported in Tables 3, and 4 to be included in table 2. Table 2 p-values reported before f-value, please rearrange.*

**Reply: Because the data in table 3 represent the "Comparison between measurements in each treatment group," and the data in table 4 show the "Comparison between each measurement interval among treatment groups," it is difficult to combine the reported data in tables 3 and 4 to be included in table 2. In all tables, the P and F values were rearranged in the same way.**

*It is suggested to measure the effect size of different interventions as significant differences did not reflect clinically significant, as the effect size is one of the most important indicators of clinical significance.*

**Reply: Yes, the effect size is one measure of clinical significance, but in our statistics, we calculated P and F-values, which represent the significant difference between interventions.**

*Discussion:*

*Paragraph 4; I suggest deleting this statement “In multiple studies, PEMFs have been shown to considerably reduce pain and enhance the quality of life in individuals with primary OP (Spadaro et al., 2011).  As the author did not evaluate or measures pain and quality of life*

**Reply: The statement has been removed from the paragraph.**

# Peer review: Reviewer response for version 1

**DOI:** 10.5256/f1000research.58012.r90355

**Published:** 2021-08-09

**Authors:** Hesham Galal Mahran

**Affiliations:** 1 Department of Physical Therapy for Surgery, Faculty of Physical Therapy, Cairo University, Giza, Egypt

This is an original research paper to evaluate the effect of full PEMF combined with exercise protocol on men with osteopenia or osteoporosis.

 The goal of this research is applicable. The title and topic are quite interesting for the journal scope. The paper is well-organized and easy to follow. Study design, methodology (materials and methods) are well described, data interpretation is appropriate and discussion is focused on the topic of the paper. The conclusion accounts for the target of the study. I really appreciate this excellent manuscript, but I have minor comments to be considered.

**Introduction section: **

- In the 3 ^rd^ paragraph, the LASER abbreviation must be defined first.

**Methods** **section:**

 - Randomization

- As the study was a double-blind study, please clarify who was blind to study; therapist and assessor or assessor and patients?

-Full-body pulsed electromagnetic field (FBPEMF) therapy

- Which waveform was employed in the PEMF application?

-Exercise training protocol

- Which type of exercise intensity was used in the study?
- Please clarify the distribution of 60 minutes between different exercise types within each session! 

**Extended data:**

- The link to the CONSORT Checklist is incorrect, check it.

Is the work clearly and accurately presented and does it cite the current literature?

Yes

If applicable, is the statistical analysis and its interpretation appropriate?

Yes

Are all the source data underlying the results available to ensure full reproducibility?

Yes

Is the study design appropriate and is the work technically sound?

Yes

Are the conclusions drawn adequately supported by the results?

Yes

Are sufficient details of methods and analysis provided to allow replication by others?

Yes

Reviewer Expertise:

Physical therapy for surgery

I confirm that I have read this submission and believe that I have an appropriate level of expertise to confirm that it is of an acceptable scientific standard.

# Response

**Published:** 2021-08-16

**Authors:** Anwar Ebid

**Affiliations:** Umm-Al-Qura University, Saudi Arabia

Dear Prof. Hesham Mahran,

 Thank you very much for your review. The authors deeply appreciate the voluntary contribution of the review in the form of valuable comments that have helped us to improve our manuscript. Every effort has been made by the authors to revise the manuscript according to your comments.

 In response to your comments, we have uploaded a newer version of this article.

 Kindly review these new versions as the revised version of this article.

 Thank you.

 Regards,

 Authors

**Introduction section: **

- In the 3 ^rd^ paragraph, the LASER abbreviation must be defined first.
- **Reply:** Light amplification by stimulated emission of radiation (LASER), it was corrected in the introduction section.

**Methods section:**

 - Randomization

- As the study was a double-blind study, please clarify who was blind to study; therapist and assessor or assessor and patients?
- **Reply:** All were blind to the study (assessor, therapist and patients)

-Full-body pulsed electromagnetic field (FBPEMF) therapy

- Which waveform was employed in the PEMF application?
- **Reply:** “Sawtooth waveforms” In clinical applications sawtooth signal is one of the most famous pulsed magnetic field shapes, whose magnetic field changes rapidly. The rapid changes in signal strength can cause large current in the tissue.

-Exercise training protocol

- Which type of exercise intensity was used in the study?

**Reply:** “Moderate intensity” This according to the previous literature which recommend it for improving the BMD, muscle and bone strength and good tolerability by the patients

- Please clarify the distribution of 60 minutes between different exercise types within each session! 
- **Reply:** Five-minute warm-up, 40 minutes initial training exercises (flexibility, aerobic exercise, strengthening, weight-bearing, and balance exercises), 10 minutes WBV and finally 5-minutes as cool-down.

**Extended data:**

- The link to the CONSORT Checklist is incorrect, check it.
- **Reply:** The correct one is: https://doi.org/10.6084/m9.figshare.14916261
